# Using the Delphi method to establish pediatric emergency triage criteria in a grade A tertiary women’s and children’s hospital in China

**DOI:** 10.1186/s12913-022-08528-8

**Published:** 2022-09-12

**Authors:** Yingying Zhao, Liqing He, Juan Hu, Jing Zhao, Mingxuan Li, Lisha Huang, Qiu Jin, Lan Wang, Jianxiong Wang

**Affiliations:** 1 Department of Emergency Nursing, West China Second University Hospital, Sichuan University/West China School of Nursing, Sichuan University, Chengdu, Sichuan China; 2 Key Laboratory of Birth Defects and Related Diseases of Women and Children (Sichuan University), Ministry of Education, Chengdu, Sichuan China

**Keywords:** Pediatric emergency department, Triage criteria, Delphi

## Abstract

**Background:**

We aimed to establish simplified and quantifiable triage criteria in pediatric emergency care, improving the efficiency of pediatric emergency triage and ensuring patient safety.

**Methods:**

We preliminarily determined the pediatric emergency triage criteria with references to pediatric emergency department characteristics and internationally recognized triage tools after literature review and discussion. The final determination of the triage criteria was reached after two rounds of Delphi surveys completed by18 experts from 3 hospitals in China.

**Results:**

Both round 1 and round 2 surveys had a 100% response rate. The overall expert authority coefficient in the two rounds of surveys was 0.872. The experts had 100% enthusiasm for participating in the surveys. Kendall’s coefficients of concordance for conditions/symptoms in patients triaged to level 1, 2, 3, and 4 were 0.149, 0.193, 0.102, and 0.266, respectively. All *p*-values were less than 0.05. The coefficients of variation in conditions/symptoms, vital signs, and the Pediatric Early Warning Score (PEWS) ranged between 0.00 and 0.205, meeting the inclusion criteria. The pediatric emergency triage criteria containing conditions/symptoms, vital signs, PEWS scores, and other 4 level 1 indicators, 51 level 2 indicators and 23 level 3 indicators were built. The maximum waiting time to treatment for the patients triaged to level 1, 2, 3, and 4 was immediate, within 10 min, within 30 min, and within 240 min, respectively.

**Conclusion:**

The pediatric emergency triage criteria established in this study was scientific and reliable. It can be used to quickly identify the patients requiring urgent and immediate care, thereby ensuring the priorities for the care of critically ill patients.

**Supplementary Information:**

The online version contains supplementary material available at 10.1186/s12913-022-08528-8.

## Background

The increasing number of pediatric emergency department (ED) patients is a common and growing problem in recent years. In the United States, children account for approximately 40% of the ED patients; In the United Kingdom, 25–30% of the accident and emergency department visits are made by children [1]. Beck et al. [2] reported that the non-urgent cases accounted for 68.4% of all cases in pediatric ED in Austria. In China, demand for pediatric emergency care has greatly exceeded the available resources and only 20% of the ED patients are in real urgent situations [3]. More attention should be paid to how to quickly identify the patients who need urgent, emergency or critical care so as to improve the efficiency of critical care. Pediatric emergency triage criteria have been widely studied and applied in developed countries such as Canada, United States, Australia and the United Kingdom. China introduced the concept of triage in the 1990s and has established the 4-level adult ED triage system (level 1: resuscitation; level 2: emergent; level 3: urgent; level 4: less urgent or non-urgent) through the following stages: making triage decision based on triage nurse’s knowledge and experience, making triage decision based on triage tool, indigenization of foreign triage tool, and development of local triage system, thereby making China’s triage criteria well in line with the international criteria. Pediatric emergency triage has become a hot topic in recent years. Shen et al. [4] proposed pediatric emergency triage criteria in 2018 based on the criteria for adults. Yang et al. [5] established a 5-level triage system for pediatric ED with reference to paedCTAS. Hu et al. [6] formulated a 5-level pediatric emergency triage criteria and a triage process undertaken by two nurses in Shanghai, China by referring to Emergency Severity Index (ESI), Australasian Triage Scale (ATS) and the Canadian Triage Acuity Scale (CTAS). These triage tools have good reliability in application, but they were established in certain areas or a specific hospital, so they may not be the optimal choice for all hospitals. We aimed to establish the pediatric emergency triage criteria suitable for our hospital based on Delphi method and the internationally recognized triage tools such as ESI and Canadian Triage and Acuity Scale Paediatric Guidelines (PaedCTAS), thereby providing a reference for the establishment of pediatric emergency triage criteria suitable for other hospitals.

## Methods

### Ethics approval

This study was conducted in accordance with the Declaration of Helsinki. All research methods were carried out in accordance with the relevant guidelines and regulations. This study was approved by the Medical Ethics Committee of West China Second University Hospital, Sichuan University [No.: YXKY2022LSP(018)]. Verbally informed consent was obtained from all participants. Medical Ethics Committee of West China Second University Hospital, Sichuan University approved the procedure of obtaining verbal informed consent.

### Research group

The research group was composed of 10 researchers with rich experience in nursing management, diagnosis and treatment of pediatric ED patients, and emergency nursing. Of them, 3 had senior professional titles and 7 had intermediate professional titles; 2 had doctoral degrees and 8 had master’s degrees. The researchers reviewed literature, translated ESI, designed the expert consultation questionnaires, and analyzed the experts’ responses.

### Literature search strategy

We searched literature in PubMed, China National Knowledge and Infrastructure (CNKI), Wanfang, and VIP Chinese Science and Technology Periodicals Full-Text Database using the following English search terms: (pediatric triage) AND (scale OR index OR system OR assessment) AND (emergency department or emergency room or emergency OR settings OR care or nursing) and the following Chinese search terms: (Er Ke Fen Zhen) AND (Ji Zhen) AND (Liang Biao OR Xi Tong OR Mo Xing OR Biao Zhun). The search time range was from January 1, 2017 to December 31, 2021. A total of 1012 articles were found. After removing duplicate records and reading the titles, abstracts, and full texts, 15 articles were finally included in this study.

### Expert consultation questionnaire

The research group preliminarily determined the pediatric emergency triage criteria with references to pediatric emergency department characteristics and internationally recognized triage tools, such as ESI and PaedCTAS after literature review and research group discussion. The preliminarily established criteria involved triage level, conditions/symptoms, vital signs, Pediatric Early Warning Score (PEWS), and the maximum waiting time to treatment for pediatric ED patients triaged to level 1 to 4. The round 1 expert consultation questionnaire contained 3 parts: (1) *Letter of instruction*. The letter of instruction outlined research background, purpose of the questionnaire, and instructions for filling the questionnaire; (2) *Information about the expert*: gender, age, professional title, educational background, service length in healthcare, service length in ED, judgement basis for the consultation items, and degree of familiarity with the items; (3) *Expert’s opinions*. The Expert’s Opinions part contained 4 forms regarding conditions/symptoms, vital signs, PEWS scores, and the maximum waiting time to treatment for pediatric ED patients. The experts were required to use 5-point Likert scale to rate the importance of the items and provide their comments on amendments and addition of new items.

### Expert selection

A total of 18 experts in nursing, diagnosis, treatment, and nursing management from the pediatric EDs of 3 national, provincial and municipal levels of grade A tertiary women’s and children’s hospitals in China were selected to participate in the consultation. Inclusion criteria: (1) Possessed an undergraduate qualification or above; (2) Had an intermediate professional title or above; (3) Had more than 10 years of clinical work experience and more than 5 years of work experience in pediatric ED; and (4) Informed of the purpose of the consultation and participated in the consultation on a voluntary basis.

### Data collection

The 2 rounds of expert consultation were conducted using e-mail. The researchers analyzed the experts’ responses after all experts returned the completed questionnaires in round 1. According to statistical requirements of the Delphi method, expert authority coefficient (Cr) ≥ 0.7 indicated a reliable consulting result and recognized the expert authority; Kendall’s coefficient of concordance was used to measure the agreement of the experts’ opinions; and the item was included when the mean score for item importance was greater than 3.5 and the coefficient of variation (CV) was lower than 0.25 [7]. For the indicators that the experts proposed amendments on, the researchers decided whether to accept the experts’ amendments after fully verifying the literature. In round 2, the round 2 questionnaire and the summary of the experts’ responses in round 1 were sent to the experts. The researchers analyzed the experts’ responses again after all experts returned the completed questionnaires in round 2. The expert consultation ended after the 2 rounds of surveys because the experts’ opinions tended to converge between round 1 and 2.

### Statistical methods

After a researcher input data in SPSS23.0, a second researcher checked data entry to ensure accurate data entry. Measurement data were expressed as mean ± standard deviation. Enumeration data were expressed as frequency and percentage. Experts’ enthusiasm to participate in the consultation was evaluated by questionnaire response rate. Degrees of authority for experts and agreement of the experts’ opinions were presented with expert authority coefficient (Cr) and Kendall’s coefficient of concordance (W), respectively. Experts’ opinions on the items were evaluated by the mean score for item importance and the CV.

## Results

### Literature search

**Fig. 1:**
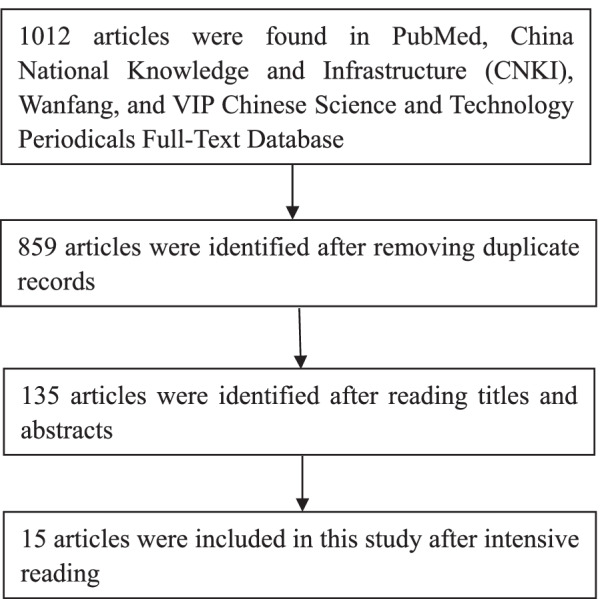
Literature selection process

A total of 1012 articles were found. Of them, 752 were in English and 260 were in Chinese. After selection, 15 articles were included. Literature selection process is shown in Fig. 1.

### Information of the experts

Information about the experts is shown in Table 1.

**Table 1:** Information about the experts (*n* = 18)

Item		n	Percentage (%)	Mean ± Standard deviation
Gender	Male	1	5.6	
	Female	17	94.4	
Age				39.50 ± 6.784
Professional title	Supervising nurse	12	66.7	
	Associate senior nurse	3	16.7	
	Senior nurse	1	5.6	
	Attending physician	1	5.6	
	Associate chief physician	1	5.6	
Educational background	Bachelor’s degree	10	55.6	
	Master’s degree	6	11.1	
	Doctoral degree	2	33.3	
Service length in healthcare				17.28 ± 7.103
Service length in pediatric emergency department				13.11 ± 5.167
Field	Nursing management	10	55.6	
	Clinical nursing	6	33.3	
	Other	2	11.1	

### Expert reliability

#### Expert enthusiasm

Both of the questionnaire response and validity rates in round 1 and 2 were 100%, indicating a high degree of enthusiasm to participate in the consultation.

#### Expert authority

Expert authority coefficient (Cr) was determined by 2 factors: the expert’ judgement basis on the consultation items (Ca) and the expert’ degree of familiarity with the items (Cs). (Cr) = (Ca + Cs)/2. The expert authority coefficient in the two rounds of consultation (Cr) = 0.872, greater than 0.7, indicating a high degree of expert authority.

### Agreement among expert opinions

The overall Kendall’s coefficients of concordance in round 1 and 2 were 0.241 and 0.164, respectively, *p* < 0.05. In round 1, Kendall’s coefficients of concordance on conditions/symptoms in the patients triaged to level 1, 2, 3, 4 were 0.300, 0.247, 0.100, and 0.152, respectively; statistically significant differences were identified in triage level 1, 2, and 4 by *p* < 0.05, but no statistically significant difference was identified in level 3 (*p* > 0.05); Kendall’s coefficient of concordance on vital signs was 0.267 (*p* < 0.05), indicating a statistically significant difference. In round 2, Kendall’s coefficients of concordance on conditions/symptoms in the patients triaged to level 1, 2, 3, 4 were 0.149, 0.193, 0.102, and 0.266, respectively, and statistically significant differences were identified by *p* < 0.05; Kendall’s coefficient of concordance on vital signs was 0.156 (*p* < 0.05), indicating a statistically significant difference (Tables 2 and 3). PEWS scores for triage level 1 to 4 in round 1 and for triage level 1 in round 2 had 94.4% agreement, but that for triage level 2 to 4 in round 2 had 100% agreement.

**Table 2:** Agreement of experts’ opinions on conditions/symptoms in patients triaged to level 1 to 4

Triage	Round 1 expert consultation			Round 2 expert consultation		
	Kendall’s	χ^2^	*p*	Kendall’s	χ^2^	*p*
Level 1	0.300	54.011	0.000	0.149	26.750	0.003
Level 2	0.247	39.959	0.000	0.193	55.548	0.000
Level 3	0.100	14.343	0.073	0.102	22.021	0.037
Level 4	0.152	19.150	0.008	0.266	9.579	0.008

**Table 3:** Agreement of experts’ opinions on vital signs

Vital signs	Kendall’s	χ^2^	*p*
Round 1 expert consultation	0.267	105.638	0.000
Round 2 expert consultation	0.156	61.585	0.000

### Degree of concentration of expert opinions

Degree of concentration of expert opinions was calculated by mean score, standard deviation, and CV of item importance. A high mean score with a low CV indicated a high level of importance. The item was included when the mean score for item importance was greater than 3.5 and the CV was lower than 0.25 [7]. The indicators removed in round 1 consultation were “the patient was stable and had mild symptoms” (CV = 0.326) and “Prescribing drugs/prescribing tests/issuing hospital admission letter to the patients who were in convalescence or the asymptomatic patients” (CV = 0.575). The indicator that was removed based on expert opinions and research group discussion was acute haemorrhage (CV = 0.225); The modified indicators were as follows: status epilepticus, convulsion, capillary refill time, and adjusting triage level in case that the patient had a history of hyperpyretic convulsion; The 15 added items were as follows: complex or multiple trauma, most severe or large burns, ocular trauma with eyeball injury, osteofascial compartment syndrome, precipitously birth (umbilical cord was not cut or Apgar score ≤ 3), incomplete airway obstruction, esophageal foreign body, severe anemia (30–60 g/L), abdominal pain (suspected strangulated intestinal obstruction, incarcerated hernia, intussusception, gastrointestinal perforation, or urinary tract calculi) with the pain score > 6, hypersomnia (able to wake up; fall asleep without stimuli) with unstable vital signs, acute asthma with stable blood pressure and pulse rate, active bleeding (epistaxis, hematuria, hematochezia, hemoptysis, or hematemesis) with unstable vital signs, unexplained abdominal distension with mental malaise, and mucocutaneous hemorrhage/platelet ≤ 20 × 10^9/L; The mean scores of importance of other items ranged between 4.17 and 5, and the CV ranged between 0 and 0.224, lower than 0.25. After round 1 consultation, 4 level 1 indicators, 51 level 2 indicators, and 23 level 3 indicators entered round 2 consultation. After round 2 consultation, each indicator’s CV was lower than 0.25, indicating convergence and high degree of concentration of expert opinions.

### Maximum waiting time for treatment

Maximum waiting time to treatment for pediatric ED patients triaged to level 1 to 4 were screened according to the degree of agreement. The maximum waiting time (level 1—immediate treatment, level 2—within 10 min, level 3—within 30 min, and level 4—within 24 min) with the highest degree of agreement was consulted in round 2 expert consultation (Table 4).

**Table 4:** Maximum waiting time to treatment for pediatric emergency department patients triaged to level 1 to 4

Triage	Maximum waiting time for treatment (min)	Degree of expert agreement
Level 1	0	100%
Level 2	5	11.1%
	10	66.7%
	15	22.2%
Level 3	20	16.7%
	30	55.5%
	60	27.8%
Level 4	90	22.2%
	150	27.8%
	240	50%

### Establishment of pediatric emergency triage criteria

Based on literature analysis and Delphi expert consultation, the pediatric emergency triage criteria was established, including conditions/symptoms, vital signs, PEWS score, 4 level 1 indicators, 51 level 2 indicators, and 23 level 3 indicators (Table 5).

**Table 5:** Pediatric emergency triage criteria established in this study

Triage	Indicators	Description	Value	Maximum waiting time for treatment
Level 1	Conditions/symptoms (critical)	Sudden cardiac arrest, respiratory arrest; Airway obstruction or asphyxia; Emergency endotracheal intubation/tracheotomy is required; Signs of shock; Sudden loss of consciousness; Signs of cerebral hernia; Life-threatening acute poisoning; Precipitously birth (umbilical cord was not cut or Apgar score ≤ 3); Complex or multiple trauma; Most severe or large burns; Ocular trauma with eyeball injury		Immediate
	Vital signs	Temperature (℃) Oxygen saturation (SpO2) AVPU (alert, verbal, pain, unresponsive) scale	≤ 35 or ≥ 41 < 90% U	
	PEWS score	PEWS ≥ 5		
	Other	The triage nurse believed that the patients was encountering a life-threatening situation and requiring emergency care		
Level 2	Conditions/symptoms (high risk)	Chest distress, chest pain, heart palpitations, stable vital signs, high risk or potential risk; Status epilepsy; Convulsion; Diabetic ketoacidosis; Acute asthma with stable blood pressure and pulse rate; Capillary refill time ≥ 3 s; Low reaction to mental state and high level of irritability; Hypersomnia (able to wake up; fall asleep without stimuli) with unstable vital signs; Newborns with temperature of > 38℃; Acute poisoning but does not meet level 1 criteria; Sudden change in consciousness; Incomplete airway obstruction; Esophageal foreign body; Severe anemia (no active bleeding) 30-60 g/L; Abdominal pain (suspected strangulated intestinal obstruction, incarcerated hernia, intussusception, gastrointestinal perforation, or urinary tract calculi) with the pain score > 6; Osteofascial compartment syndrome; Active bleeding (epistaxis, hematuria, hematochezia, hemoptysis, or hematemesis) with unstable vital signs		< 10 min
	Vital signs	Pulse rate (beats/min)	*P* > 180 (y < 3 months old); *P* > 160 (3 months old ≤ y < 3 years old); *P* > 140 (3 years old ≤ y < 8 years old); *P* > 100 (y ≥ 8 years old)	
		Respiration rate (breaths/min)	*R* > 50 (y < 3 months old); *R* > 40 (3 months old ≤ y < 3 years old); *R* > 30 (3 years old ≤ y < 8 years old); *R* > 20 (y ≥ 8 years old)	
		SpO2	90% ~ 92%	
		Systolic blood pressure	> 130 mmHg (≥ 5 years old) or < 75 mmHg (≥ 5 years old)	
	PEWS score	PEWS = 3 ~ 4		
	Other	The triage nurse believed that the patients was at a high-risk situation or potential risk but required no emergency care		
Level 3	Conditions/symptoms	Intermittent epileptic seizures; With a history of hyperpyretic convulsion; Foreign body aspiration but no breathing difficulty; Dysphagia but no breathing difficulty; Mental and behavior disorder; Severe vomiting; Symptoms of allergic reaction (obvious rashes on the skin and mucous membranes, extensive facial swelling, etc.); Hypersomnia (able to wake up; fall asleep without stimuli) with stable vital signs; Moderate to severe pain with any cause (score: 4–6); Stable newborns; Active bleeding (epistaxis, hematuria, hematochezia, hemoptysis, or hematemesis) with stable vital signs; Unexplained abdominal distension with mental malaise; Mucocutaneous hemorrhage/platelet ≤ 20 × 10^9/L		< 30 min
	Vital signs	Pulse rate (beats/min)	88 < *P* < 180 (y < 3 months old); 80 < *P* < 160 (3 months old ≤ y < 3 years old); 64 < *P* < 140 (3 years old ≤ y < 8 years old); 56 < *P* < 120 (y ≥ 8 years old)	
		Respiration rate (breaths/min)	24 < *R* < 50 (y < 3 months old); 20 < *R* < 40 (3 months old ≤ y < 3 years old); 16 < *R* < 30 (3 years old ≤ y < 8 years old); 14 < *R* < 24 (y ≥ 8 years old)	
	PEWS score	PEWS = 1 ~ 2		
	Other	The pediatric patient had acute symptoms and emergency issues		
Level 4	Conditions/symptoms	Vomiting or diarrhea without dehydration; Mild pain		< 240 min
	PEWS score	PEWS = 0		
	Other	Mild or non-urgent condition		

## Discussion

### Significance of establishing pediatric emergency triage criteria

Some pediatric diseases are seasonal and pediatric patients generally have sudden onset symptoms. A large number of pediatric patients come to the outpatient department for treatment during the high-incidence season. Because there are limited places for outpatient appointments or no outpatient services are provided at night, the pediatric patients who can not visit the outpatient department visit the pediatric ED, thereby causing ED crowding. The higher the triage level the patient is at, the higher the hospitalization rate, more extended periods of hospitalization, and higher mortality that the patient may have [8]. ED crowding and under-detection of an urgent category of priority need can prolong the waiting time for treatment, decrease the satisfaction level of the pediatric patients and their family members, and result in severe consequences for the patients. Our hospital is a national grade A tertiary women’s and children’s hospital. There were approximately 200000 pediatric ED visits in our hospital in 2021, but only 9.98% of them were made by critically ill patients. The limited medical resources cannot meet the needs of all patients, therefore it is important to establish an efficient and sensitive pediatric emergency triage criteria. Triage is the first step during ED visit and also an important factor influencing the quality of nursing. Pediatric ED patients not only have a wide scope of age ranges and different types of conditions, they may also cry easily and have poor expression, which may make triage difficult. How to quickly complete the assessment on the pediatric ED patient’s conditions and make an optimal decision within limited time is a problem that need to be solved [9]. The pediatric emergency triage criteria established based on Delphi method are objective and quantifiable.

### Scientificity and reliability

The pediatric emergency triage criteria established in this study was in accordance with the guidelines of National Health Commission of China and with references to pediatric emergency department characteristics and internationally recognized triage tools, such as ESI and PaedCTAS after literature review, research group discussion, and 2 rounds of expert consultation. The study design was scientific and rigorous, and the statistical methods were correct.

In this study, the experts in clinical nursing, diagnosis, treatment, and nursing management from the pediatric EDs of 3 grade A tertiary women’s and children’s hospitals at national, provincial and municipal levels participated in the consultation. The Delphi expert panel is generally composed of 15–30 experts. A total of 18 experts participated in our consultation, ensuring the reliability of this study. The overall expert authority coefficient in the 2 rounds of surveys was 0.872, greater than 0.7, indicating a high degree of expert authority. All indicator’s CVs after round 2 consultation were lower than 0.25, showing that the triage criteria established in this study were highly recognized by the experts.

### Analysis of indicators

Emergency triage is a process of rapid assessment and determination of medical priority and arrangement of medical visits based on signs of the patients and chief complaints provided by the patients’ family members [10]. Emergency triage criteria is used to triage the ED patients into different levels according to the common signs and symptoms of patients, giving priorities for critically ill patient [11]. The triage nurses can use the triage criteria to make targeted evaluation on patients conditions in a timely manner.

A total of 43 indicators about conditions/symptoms were determined after 2 rounds of expert consultation. Of them, 11 were level 1 indicators (critical), 17 were level 2 indicators (high risk), 13 were level 3 indicators, and 2 were level 4 indicators. The indicators about conditions/symptoms involved 4 factors: airway, breathing, circulation, and consciousness. Previous studies [12, 13] have shown that triage nurses can identify the pediatric ED patients who need critical or urgent care by triaging the patients to different levels according to the patients’ conditions and symptoms, thereby ensure patient safety. Some of the indicators about conditions/symptoms in this study were from that for adults [14], and the classification of triage in this study was in consistent with that of ESI [15]. ESI which is an effective tool for pediatric emergency triage is accurate and reliable [16, 17].

### Indicators about vital signs

A total of 23 indicators about vital signs were determined after expert consultation. Many triage nurses in China evaluate patient conditions based on their own intuition and experience, not a standardized objective indicators. Vital signs have been proved to be an important part in triage assessment [18] and an important predictor of disease progression [19]. The results of Zachariasse et al. [18] have shown that the determining cut-off values of vital signs for patients of different age groups can help nurses triage the patients. However, Zachariasse et al. did not elaborate on age group classification. Because of age differences, low specificity of the indicators on vital signs, and low sensitivity of the indicator on blood pressure, early identification and intervention of critically ill patients may be complicated in triage [20, 21], so a further research on sensitivity and specificity of the indicators is needed.

### PEWS

PEWS is a commonly used assessment tool for the severity of pediatric patients. It can quickly and early identify critically ill patients at a early stage and provide a reference for emergency triage and determination of admission to the intensive care unit. Lillitos et al. [22] reported that PEWS had low sensitivity (32%, 44%) in predicting hospital admission and severe diseases when it was used in emergency triage, indicating that PEWS might be unsuitable for use as a triage standard alone. McElroy et al. [23] pointed out that combined use of PEWS and CTAS could identify the early signs of deterioration and improve the nursing value of pediatric triage. Therefore, our study included PEWS in the triage system as a tool to identify the patients’ potential risks.

### Maximum waiting for treatment

Maximum waiting time to treatment for pediatric ED patients is critical during ED visits. The short waiting time generally lead to a better medical outcome. To decrease the waiting time, the patients who meet level 1 criteria shall be immediately sent to the resuscitation room without further evaluation based on vital signs and PEWS [24], then, then level 2 patients can be treated within short waiting time. The level 3 patients may rapidly develop into level 2 or 1, requiring close monitoring [25]. Lin et al. [26] reported that 25% of level patients waiting for treatment experienced changes in their condition, indicating the importance of decreasing waiting time. Hinson et al. [27] and Roquette et al. [28] also described the waiting time: level 1—immediate, level2—within 10 min, level 3—within 30 min, and level 4—within 240 min.

### Limitations

All of the experts in this study were from Chengdu. Expert representativeness might be weakened because the experts were not from different cities or provinces. In addition, no clinical studies was carried out since the establishment of the triage criteria in our study. Reliability and validity of our triage criteria needs empirical analysis. Therefore, an empirical study of pediatric emergency triage criteria is needed.

## Conclusions

The pediatric emergency triage criteria established in this study was scientific and reliable. It can be used to quickly identify the patients requiring urgent and immediate care, thereby ensuring the priorities for the care of critically ill patients. However, there is a wide variety of diseases in pediatric ED patients and the established triage criteria cannot contain all conditions. Triage nurses should quickly judge the patients’ conditions based on triage criteria as well as work experience to ensure patient safety.

## Supplementary Information

**Additional file 1. **

## Data Availability

The datasets used and/or analysed during the current study are not publicly available because the participants’ personal information was included in this study, but the data that were not involved the participants’ personal information are available from the corresponding author on reasonable request.

## Abbreviations

ATS: Australasian Triage Scale
AVPU: Alert, Verbal, Pain, Unresponsive
CTAS: Canadian Triage and Acuity Scale
CV: Coefficient of variation
ED: Emergency Department
ESI: Emergency Severity Index
PaedCTAS: Canadian Triage and Acuity Scale Paediatric Guidelines
PEWS: Pediatric Early Warning Score
SpO2: Oxygen Saturation
