# Supplementary material for: Using the Delphi method to establish pediatric emergency triage criteria in a grade A tertiary women’s and children’s hospital in China
Source: BMC Health Serv Res. 2022 Sep 12;22:1154. doi: 10.1186/s12913-022-08528-8 (PMC9469547; doi:10.1186/s12913-022-08528-8)
Supplement: Supplementary file 1 — Additional file 1. [file 12913_2022_8528_MOESM1_ESM.docx]

**Part 1** Conditions/symptoms in the two rounds of expert consultation

| 1^st^ round | | | | | 2^nd^ round | |
| --- | --- | --- | --- | --- | --- | --- |
| Conditions/symptoms | | Mean ± Standard deviation | CV | Decision | Mean ± Standard deviation | CV |
| Level 1 | Sudden cardiac arrest and respiratory arrest | 5.00 ± 0.000 | 0.000 | Retained | 5.00 ± 0.000 | 0.000 |
|  | Airway obstruction or asphyxia | 5.00 ± 0.000 | 0.000 | Retained | 5.00 ± 0.000 | 0.000 |
|  | Emergency endotracheal intubation/tracheotomy is required | 5.00 ± 0.000 | 0.000 | Retained | 5.00 ± 0.000 | 0.000 |
|  | Signs of shock | 4.89 ± 0.323 | 0.066 | Retained | 4.89 ± 0.323 | 0.066 |
|  | Sudden loss of consciousness | 4.94 ± 0.236 | 0.048 | Retained | 4.89 ± 0.323 | 0.066 |
|  | Status epilepsy (the experts suggested adding status epilepsy to Level 2) | 4.72 ± 0.575 | 0.122 | Put in Level 2 | 4.94 ± 0.236 | 0.048 |
|  | Signs of cerebral hernia | 4.94 ± 0.236 | 0.048 | Retained | 4.83 ± 0.383 | 0.079 |
|  | Life-threatening acute poisoning | 4.83 ± 0.383 | 0.079 | Retained | 4.89 ± 0.323 | 0.066 |
|  | Convulsion | 4.28 ± 1.320 | 0.308 | Put in Level 2 | 4.78 ± 0.732 | 0.153 |
|  | Acute massive haemorrhage | 4.61 ± 1.037 | 0.225 | Removed |  |  |
|  | Capillary refill time ≥3 seconds | 4.11 ± 1.278 | 0.311 | Put in Level 2 | 4.56 ± 0.616 | 0.135 |
|  | Precipitously birth (umbilical cord was not cut or Apgar score ≤ 3) |  |  | Added as a new indicator | 4.94 ± 0.236 | 0.048 |
|  | Complex or multiple trauma |  |  | Added as a new indicator | 4.72 ± 0.461 | 0.098 |
|  | Most severe or large burns |  |  | Added as a new indicator | 4.89 ± 0.323 | 0.066 |
|  | Ocular trauma with eyeball injury |  |  | Added as a new indicator | 4.67 ± 0.485 | 0.104 |
| Level 2 | Chest distress, chest pain, heart palpitations, stable vital signs, high risk or potential risk | 4.61 ± 0.698 | 0.151 | Retained | 4.83 ± 0.383 | 0.079 |
|  | With a history of hyperpyretic convulsion | 3.83 ± 1.295 | 0.338 | Put in Level 3 | 4.56 ± 0.616 | 0.135 |
|  | Low reaction to mental state and high level of irritability | 4.28 ± 0.958 | 0.224 | Retained | 4.39 ± 0.778 | 0.177 |
|  | Newborns with temperature of > 38℃ | 4.17 ± 0.857 | 0.206 | Retained | 4.39 ± 0.608 | 0.138 |
|  | Acute poisoning but does not meet level 1 criteria | 4.44 ± 0.784 | 0.177 | Retained | 4.44 ± 0.616 | 0.139 |
|  | Sudden change in consciousness | 4.72 ± 0.575 | 0.122 | Retained | 4.78 ± 0.428 | 0.090 |
|  | Diabetic ketoacidosis | 4.44 ± 0.984 | 0.222 | Retained | 4.56 ± 0.511 | 0.112 |
|  | Acute asthma with stable blood pressure and pulse rate |  |  | Added as a new indicator | 4.67 ± 0.485 | 0.104 |
|  | Hypersomnia (able to wake up; fall asleep without stimuli) with unstable vital signs |  |  | Added as a new indicator | 4.78 ± 0.428 | 0.090 |
|  | Incomplete airway obstruction (added according to the experts’ suggestion) |  |  | Added as a new indicator | 4.67 ± 0.485 | 0.104 |
|  | Esophageal foreign body |  |  | Added as a new indicator | 4.39 ± 0.608 | 0.138 |
|  | Severe anemia (no active bleeding) 30-60g/L |  |  | Added as a new indicator | 4.61 ± 0.608 | 0.131 |
|  | Abdominal pain (suspected strangulated intestinal obstruction, incarcerated hernia, intussusception, gastrointestinal perforation, or urinary tract calculi) with the pain score > 6 |  |  | Added as a new indicator | 4.56 ± 0.511 | 0.112 |
|  | Osteofascial compartment syndrome |  |  | Added as a new indicator | 4.44 ± 0.705 | 0.159 |
|  | Active bleeding (epistaxis, hematuria, hematochezia, hemoptysis, or hematemesis) with unstable vital signs |  |  | Added as a new indicator | 4.94 ± 0.236 | 0.048 |
| Level 3 | Intermittent epileptic seizures | 4.72 ± 0.575 | 0.122 | Retained | 4.67 ± 0.485 | 0.104 |
|  | Foreign body aspiration but no breathing difficulty | 4.44 ± 0.705 | 0.159 | Retained | 4.72 ± 0.461 | 0.098 |
|  | Dysphagia but no breathing difficulty | 4.28 ± 0.958 | 0.224 | Retained | 4.44 ± 0.784 | 0.177 |
|  | Severe vomiting | 4.61 ± 0.778 | 0.169 | Retained | 4.67 ± 0.485 | 0.104 |
|  | Symptoms of allergic reaction (obvious rashes on the skin and mucous membranes, extensive facial swelling, etc.) | 4.44 ± 0.856 | 0.193 | Retained | 4.67 ± 0.485 | 0.104 |
|  | Hypersomnia (able to wake up; fall asleep without stimuli) with stable vital signs | 4.17 ± 0.857 | 0.206 | Retained | 4.56 ± 0.511 | 0.112 |
|  | Moderate to severe pain with any cause (score: 4-6) | 4.61 ± 0.608 | 0.132 | Retained | 4.72 ± 0.461 | 0.098 |
|  | Stable newborns | 4.72 ± 0.575 | 0.122 | Retained | 4.33 ± 0.840 | 0.194 |
|  | Active bleeding (epistaxis, hematuria, hematochezia, hemoptysis, or hematemesis) with stable vital signs |  |  | Added as a new indicator | 4.61 ± 0.502 | 0.109 |
|  | Unexplained abdominal distension with mental malaise |  |  | Added as a new indicator | 4.78 ± 0.428 | 0.090 |
|  | Mucocutaneous hemorrhage/platelet ≤ 20×10^9/L |  |  | Added as a new indicator | 4.72 ± 0.575 | 0.122 |
| Level 4 | Vomiting or diarrhea without dehydration | 4.22 ± 0.943 | 0.223 | Retained | 4.67 ± 0.686 | 0.147 |
|  | Mild pain | 4.44 ± 0.984 | 0.222 | Retained | 4.50 ± 0.924 | 0.205 |
|  | Stable condition with mild symptoms | 3.83 ± 1.249 | 0.326 | Removed |  |  |
|  | Prescribing drugs/prescribing tests/issuing hospital admission letter to the patients who were in convalescence or the asymptomatic patients | 3.17 ± 1.823 | 0.575 | Removed |  |  |

**Part 2** Vital signs in the two rounds of expert consultation

| 1^st^ round of expert consultation | | | | | 2^nd^ round of expert consultation | |
| --- | --- | --- | --- | --- | --- | --- |
| Vital signs |  | Level | Mean ± Standard deviation | CV | Mean ± Standard deviation | CV |
| Heart rate (beats/min) | P > 180 (y < 3 months old) | 2 | 4.67 ± 0.485 | 0.103 | 4.83 ± 0.383 | 0.079 |
|  | P > 160 (3 months old < y < 3 years old) | 2 | 4.44 ± 0.784 | 0.177 | 4.83 ± 0.383 | 0.079 |
|  | P > 140 (3 years old < y < 8 years old) | 2 | 4.39 ± 0.778 | 0.177 | 4.56 ± 0.616 | 0.135 |
|  | P > 100 (y > 8 years old) | 2 | 4.06 ± 0.802 | 0.198 | 4.22 ± 0.808 | 0.191 |
|  | 88 < P < 180 (y < 3 months old) | 3 | 4.44 ± 0.856 | 0.193 | 4.72 ± 0.669 | 0.142 |
|  | 80 < P < 160 (3 months old < y < 3 years old) | 3 | 4.39 ± 0.850 | 0.194 | 4.67 ± 0.686 | 0.147 |
|  | 64 < P < 140 (3 years old < y < 8 years old) | 3 | 4.33 ± 0.840 | 0.194 | 4.50 ± 0.707 | 0.157 |
|  | 56 < P < 108 (y > 8 years old) | 3 | 4.17 ± 0.857 | 0.206 | 4.44 ± 0.856 | 0.193 |
| Respiration rate (breaths/min) | R > 50 (y < 3 months old) | 2 | 4.44 ± 0.616 | 0.139 | 4.83 ± 0.383 | 0.079 |
|  | R > 40 (3 months old < y < 3 years old) | 2 | 4.39 ± 0.608 | 0.138 | 4.61 ± 0.502 | 0.109 |
|  | R > 30 (3 years old < y < 8 years old) | 2 | 4.17 ± 0.857 | 0.206 | 4.44 ± 0.705 | 0.159 |
|  | R > 20 (y > 8 years old) | 2 | 4.06 ± 0.802 | 0.198 | 4.22 ± 0.943 | 0.223 |
|  | 24 < R < 50 (y < 3 months old) | 3 | 4.33 ± 0.970 | 0.224 | 4.56 ± 0.784 | 0.172 |
|  | 20 < R < 40 (3 months old < y < 3 years old) | 3 | 4.06 ± 0.938 | 0.231 | 4.61 ± 0.778 | 0.169 |
|  | 16 < R < 30 (3 years old < y < 8 years old) | 3 | 4.22 ± 0.943 | 0.223 | 4.50 ± 0.786 | 0.175 |
|  | 14 < R< 20 (y > 8 years old) | 3 | 4.44 ± 0.984 | 0.222 | 4.28 ± 0.826 | 0.193 |
| SpO2 | SpO2 < 90% | 1 | 4.78 ± 0.428 | 0.090 | 4.83 ± 0.514 | 0.106 |
|  | SpO2 (90-92%) | 2 | 4.44 ± 0.784 | 0.177 | 4.72 ± 0.461 | 0.098 |
| Temperature | ≥41℃ | 1 | 4.33 ± 0.594 | 0.137 | 4.56 ± 0.616 | 0.135 |
|  | ≤35℃ | 1 | 4.67 ± 0.485 | 0.104 | 4.83 ± 0.383 | 0.079 |
| AVPU (alert, verbal, pain, unresponsive) scale | U | 1 | 5.00 ± 0.000 | 0.000 | 4.94 ± 0.236 | 0.048 |
| Systolic blood pressure | > 130mmHg | 2 | 4.56 ± 0.511 | 0.112 | 4.50 ± 0.707 | 0.157 |
|  | < 75mmHg | 2 | 4.72 ± 0.461 | 0.098 | 4.61 ± 0.698 | 0.151 |
